# Supplementary material for: Revealing Inflammatory Indications Induced by Titanium Alloy Wear Debris in Periprosthetic Tissue by Label-Free Correlative High-Resolution Ion, Electron and Optical Microspectroscopy
Source: Materials (Basel). 2021 Jun 3;14(11):3048. doi: 10.3390/ma14113048 (PMC8199876; doi:10.3390/ma14113048)
Supplement: Supplementary file 1 [file materials-14-03048-s001.zip › materials-1224244-SI.pdf]

materials

Supplementary Materials

Revealing Inflammatory Indications Induced by Titanium Alloy Wear Debris in Periprosthetic Tissue by Label-Free Correlative High-Resolution Ion, Electron and Optical Microspectroscopy

Rok Podlipec [1,2,*], Esther Punzón-Quijorna [3,*], Luka Pirker [2], Mitja Kelemen [3], Primož Vavpetič [3], Rajko Kavalar [4], Gregor Hlawacek [1], Janez Štrancar [2], Primož Pelicon [3] and Samo K. Fokter [5]

[1] Ion Beam Center, Helmholtz-Zentrum Dresden-Rossendorf e.V., Bautzner Landstrasse 400, 01328 Dresden, Germany; g.hlawacek@hzdr.de
[2] Condensed Matter Physics Department, Jožef Stefan Institute, Jamova Cesta 39, 1000 Ljubljana, Slovenia; luka.pirker@ijs.si (L.P.); janez.strancar@ijs.si (J.Š.);
[3] Department of Low and Medium Energy Physics, Jožef Stefan Institute, Jamova Cesta 39, 1000 Ljubljana, Slovenia; mitja.kelemen@ijs.si (M.K.); primoz.vavpetic@ijs.si (P.V.); primoz.pelicon@ijs.si (P.P.)
[4] Department of Pathology, University Medical Centre Maribor, Ljubljanska ulica 5, 2000 Maribor, Slovenia; rajko.kavalar@ukc-mb.si
[5] Department of Orthopaedics, University Medical Centre Maribor, Ljubljanska ulica 5, 2000 Maribor, Slovenia; samo.fokter@guest.arnes.si
* Correspondence: r.podlipec@hzdr.de (R.P.); esther.punzon-quijorna@ijs.si (E.P.-Q.); Tel.: +386-1-477-3167 (R.P.)

1. Instrumentation of the label-free multimodal and multiscale correlative microscopy by advanced ion, electron and optical based microscopies

The experimental workflow for studying wear debris properties and related biological response in periprosthetic tissue from a broken hip prosthesis comprise the following correlative microscopy approaches:

- Figure S1—sub μm resolution optical imaging and spectroscopy using hybrid and multimodal techniques: CLSM, NIR RCM, FLIM and fHSI;
- Figure S2—sub μm resolution electron beam analysis (SEM-EDS) and nm resolution imaging (HIM);
- Figure S3—μm resolution ion beam analysis (micro-PIXE)

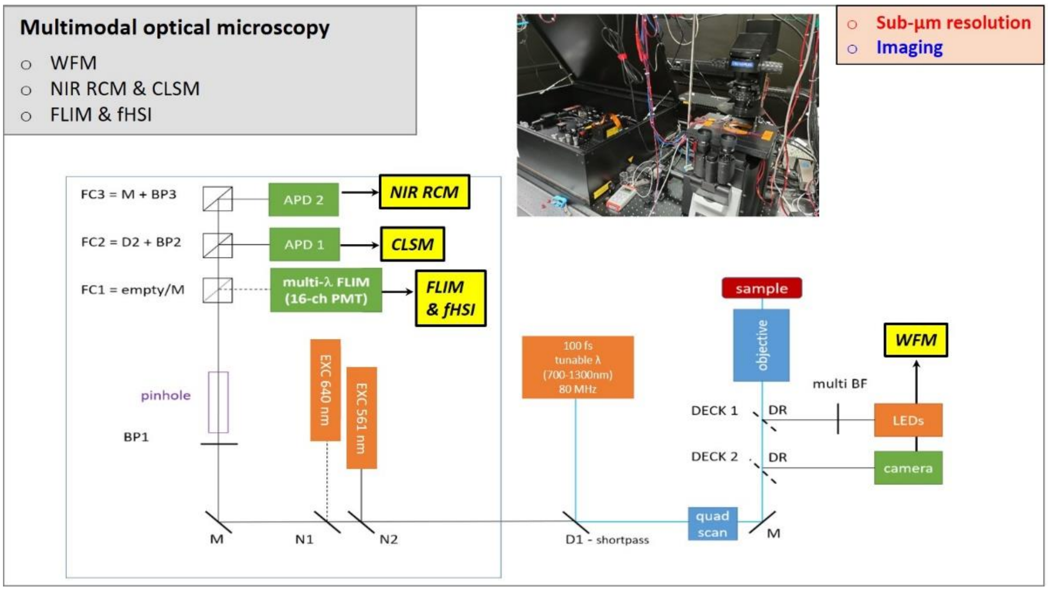

Figure S1. Instrumentation of multimodal optical microscopy to identify wear debris biological impact on the surrounding periprosthetic tissue. The presented system enables simultaneous acquisition of near-infrared reflectance confocal microscopy (NIR RCM), confocal laser scanning microscopy (CLSM), fluorescence lifetime imaging microscopy (FLIM) and fluorescence hyperspectral imaging (fHSI). The excitation sources setup (in orange): LED for wide-field fluorescence microscopy (WFM); pulsed tunable fs NIR laser (λ = 740–780nm) for NIR RCM; and pulsed diode ps laser (λ = 561 nm) for CLSM, FLIM and fHSI. The detection setup (in green): RGB camera for WFM; avalanche photodiodes APDs for NIR RCM and CLSM; and 16-Ch PMT for FLIM and fHSI. The optical component: FC—filter cube; M—mirror; BP—band pass; D—dichroic; N—notch.

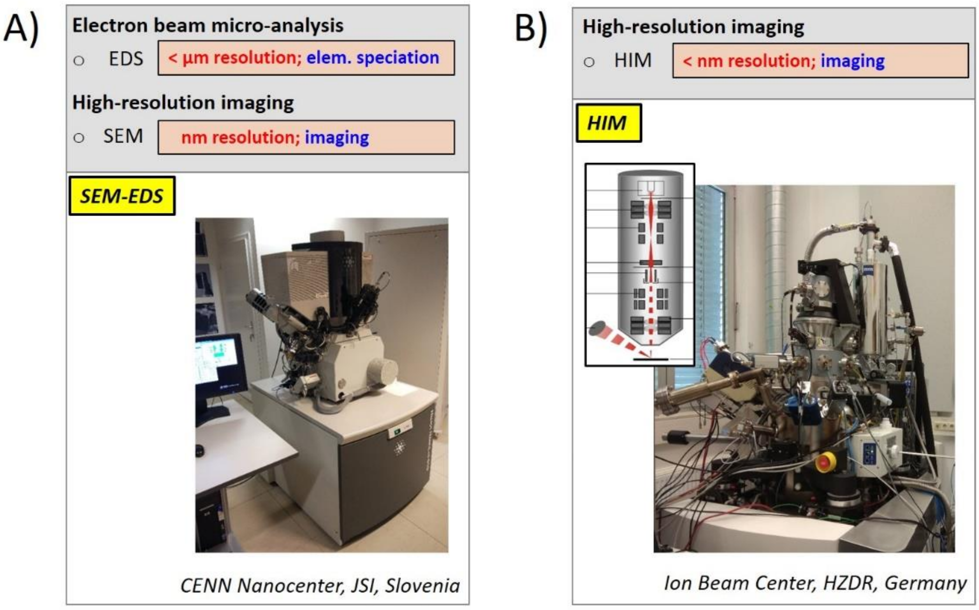

Figure S2. Scanning Electron Microscope (SEM) and Helium Ion Microscope (HIM) used for elemental analysis and nm resolution imaging of wear debris and the surrounding periprosthetic tissue. (**A**) FEI HeliosNanolab 650 SEM with energy-dispersive X-ray spectroscopy module (EDS, Oxford instruments, X-max 50 mm^2 SDD detector). The EDS spectra of samples were collected with the following experimental parameters: electron acceleration voltage (15 kV), electron current (200 pA) and chamber vacuum (10^{-6} hPa). To minimize the charging effects, samples were coated with approximately 10 nm layer of carbon; (**B**) Orion NanoFab HIM (Zeiss). Imaging by collecting

secondary electrons (SE1), was done with the following experimental parameters: He ion energy (30 keV), ion current (1.7 pA) and chamber vacuum (3×10^{-7} hPa). A scheme of the major HIM components used for beam focusing and manipulation is adopted from [1].

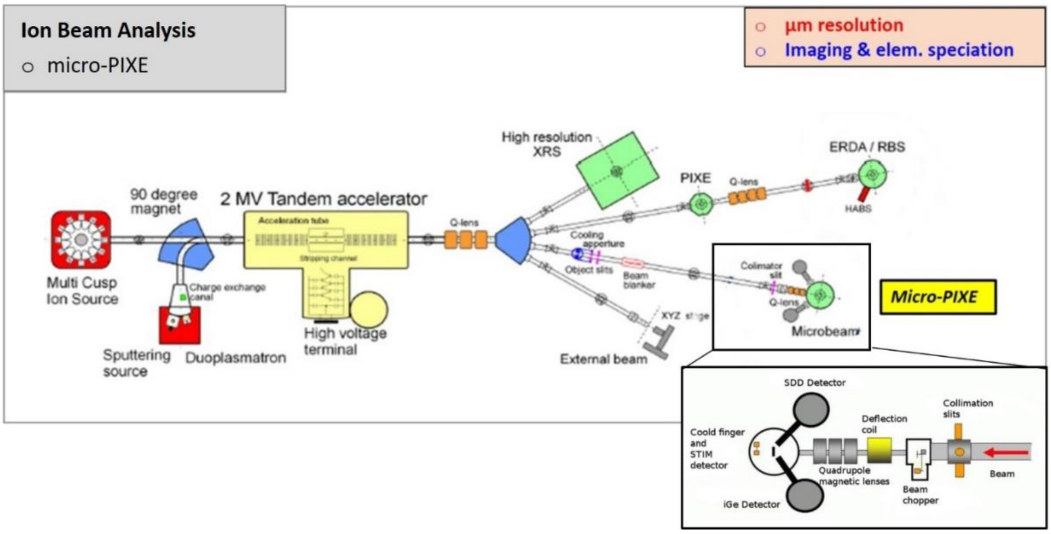

Figure S3. Microbeam set up used in micro-PIXE analysis for the elemental mapping of the wear debris in periprosthetic tissue with µm resolution (Microanalytical Center, JSI). Micro-PIXE beam line is coupled with the 2 MV TandetronTM accelerator and H- ions are produced in high brightness multicusp ion source (High Voltage Engineering Europa). The high brightness of the ion source, allows the reduction of object slit aperture and acceptance angle at the nuclear microprobe, resulting in a reduced beam size down to 500 nm, which significantly improves the lateral resolution for micro-PIXE analysis in analytical regime of operation [2].

2. FLIM and fHSI analysis of wear-debris and the impact on the surrounding tissue

By multimodal optical imaging and spectroscopy using fluorescence lifetime imaging microscopy (FLIM, Figure S4) and fluorescence hyperspectral imaging (fHSI, Figure S5), details of wear debris impact on biological properties of the surrounding tissue were revealed. Very well distinguished regions were characterized by FLIM analysis (Figure S4 A) with the corresponding FLIM decay curves (Figure S4 B) and their fitted values in the table (Figure S4C). Further hyperspectral analysis (Figure S5A), with the fluorescence spectra from distinct regions (Figure S5B) and distribution histogram of fitted spectral peaks over the field-of-view (Figure S5C) showed endogenous fluorophores accumulation. Quantification of biological molecules revealed lipofuscin presence found accumulated in lysosomes which is typically caused by an oxidative stress, in our case, the nearby wear debris.

A) B)

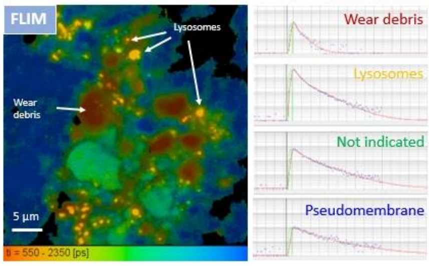

C)

Region/ Function/ Fluorescence origin	τ_1 (ps)	a_1 [%]	τ_2 (ps)	a_2 [%]
Region-1<br>Wear debris<br>LSP effect from nearby fluor.	220 ± 40	84 ± 4	1000 ± 80	16 ± 4
Region-2<br>Lysosomes<br>Lipofuscin	450 ± 50	85 ± 2	1700 ± 100	15 ± 2
Region-3<br>Not indicated<br>Other lipopigments	700 ± 50	76 ± 2	2300 ± 150	24 ± 2
Region-4<br>Pseudomembrane;<br>Not indicated	800 ± 50	51 ± 5	2700 ± 150	49 ± 5

Figure S4. FLIM analysis of wear debris and surrounding periprosthetic tissue on a sub-micron scale using double exponent decay fitting. (**A**) FLIM image of endogenous fluorophores color-coded with an average fluorescence decay time (legend below); (**B**) FLIM decay curves and best fits of the distinct structural/functional sites in the measured tissue; (**C**)

table of fitted lifetimes τ_i and their relative amplitudes ai served for quantification of the distinct sites, from wear debris to different auto-fluorescent molecules in the tissue.

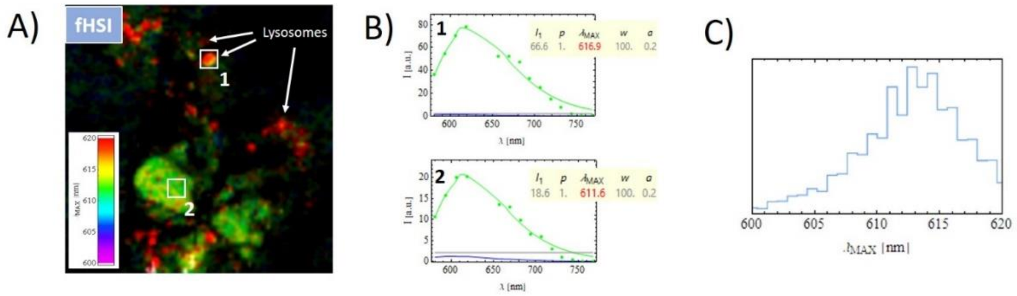

Figure S5. fHSI analysis of the periprosthetic tissue surrounding wear debris on a sub-micron scale with an empirical log-normal spectral model fitting as described before [3]. (**A**) fHSI image of endogenous fluorophores with distinct spectral peaks, e.g. lipofuscin accumulating in lysosomes; (**B**) fluorescence spectra and best fits of the two distinct regions; (**C**) Spectral peak distribution of endogenous fluorophores in the measured area.

3. HIM vs SEM high-resolution imaging on wear debris

By comparing the capability of both techniques through imaging of the same sample site, one can easily notice the outperformance of HIM (Figure S6). In contrast to SEM, HIM was capable to identify and differentiate surface topographies with the nm features (see the marked regions). Besides, much better depth contrast has been shown using HIM. This derives from pure physics principles as the shorter de Broglie wavelength in HIM enables significantly smaller probe size and less scattering on the surface as compared to SEM. Besides, no need for sample pre-coating is required in HIM due to charge compensating element in its instrumentation.

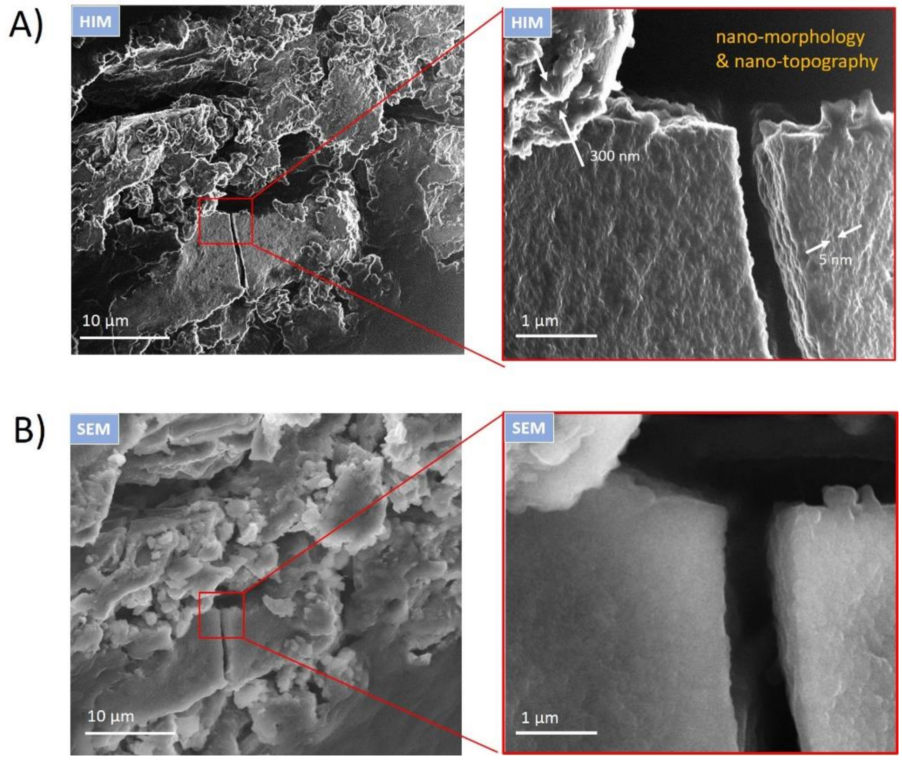

Figure S6. Comparison of the resolution, surface sensitivity and depth-of-field of (**A**) HIM and (**B**) SEM acquired on the same wear debris site in the periprosthetic tissue marked with white rectangle on Figure S8A. The experimental parameters used in HIM were 30 keV He ion energy, 1.7 pA

ion current and 2 nm pixel step size. The experimental parameters used in SEM were 15 kV for the electron source, 25 pA electron current and 2 nm pixel step size.

4. Elemental analysis of the wear debris and the surrounding tissue using SEM-EDS

The following Figure S7 and Figure S8 show detailed elemental maps and quantification of the periprosthetic tissue full of wear debris on a few hundred microns field-of-view. The region of SEM-EDS analysis shown in Figure. 4 of the main manuscript, is here denoted with a white rectangle in Figure S8A.

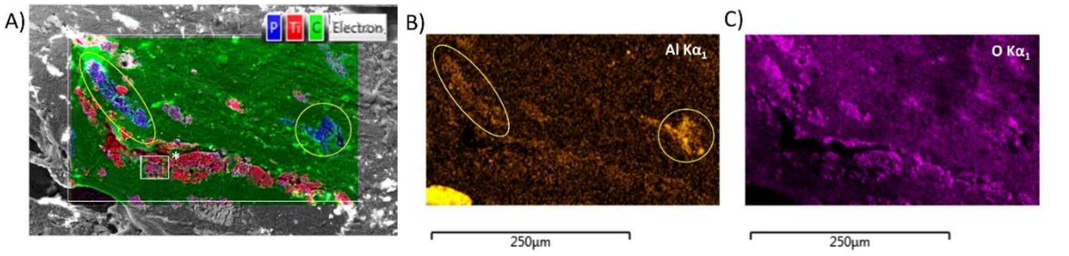

Figure S7. SEM-EDS map of the most abundant elements in the periprosthetic tissue filled with wear debris from Ti-6Al-4V alloy. (**A**) Overlay of SEM-EDS map showing elements phosphorus (P), titanium (Ti) and carbon (C); (**B**) EDS map of aluminium (Al) that coincides with regions rich in P (see the marked yellow insets) and (**C**) EDS map of oxygen (O) that coincides with regions rich in Ti.

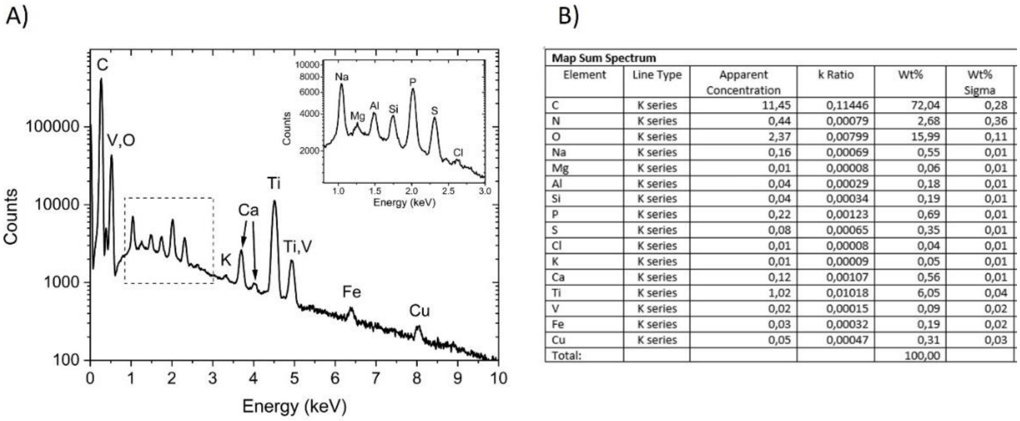

Map Sum Spectrum					
Element	Line Type	Apparent Concentration	k Ratio	Wt%	Wt% Sigma
C	K series	11,45	0,11446	72,04	0,28
N	K series	0,44	0,00079	2,68	0,36
O	K series	2,37	0,00799	15,99	0,11
Na	K series	0,16	0,00069	0,55	0,01
Mg	K series	0,01	0,00008	0,06	0,01
Al	K series	0,04	0,00029	0,18	0,01
Si	K series	0,04	0,00034	0,19	0,01
P	K series	0,22	0,00123	0,69	0,01
S	K series	0,08	0,00065	0,35	0,01
Cl	K series	0,01	0,00008	0,04	0,01
K	K series	0,01	0,00009	0,05	0,01
Ca	K series	0,12	0,00107	0,56	0,01
Ti	K series	1,02	0,01018	6,05	0,04
V	K series	0,02	0,00015	0,09	0,02
Fe	K series	0,03	0,00032	0,19	0,02
Cu	K series	0,05	0,00047	0,31	0,03
Total:				100,00	

Figure S8. M-EDS elemental quantification summed over the marked tissue region shown in the Figure S7. (**A**) EDS spectra with the inset Figure of the most abundant elements in the energy spectrum from 1keV to 3 keV and (**B**) wt% calculation of the elements in the scanned region.

References

1. Klingner, N.; Heller, R.; Hlawacek, G.; Borany, J. von; Notte, J.; Huang, J.; Facsko, S. Nanometer Scale Elemental Analysis in the Helium Ion Microscope Using Time of Flight Spectrometry. *Ultramicroscopy* **2016**, *162*, 91–97, doi:10.1016/j.ultramic.2015.12.005.
2. Pelicon, P.; Podaru, N.C.; Vavpetič, P.; Jeromel, L.; Ogrinc Potocnik, N.; Ondračka, S.; Gottdang, A.; Mous, D.J.M. A High Brightness Proton Injector for the Tandetron Accelerator at Jožef Stefan Institute. *Nucl. Instrum. Methods Phys. Res. Sect. B: Beam Interact. Mater. At.* **2014**, *332*, 229–233, doi:10.1016/j.nimb.2014.02.067.
3. Urbančič, I.; Arsov, Z.; Ljubetič, A.; Biglino, D.; Strancar, J. Bleaching-Corrected Fluorescence Microspectroscopy with Nanometer Peak Position Resolution. *Opt. Express* **2013**, *21*, 25291–25306, doi:10.1364/OE.21.025291.
